# Early Bearing Fault Diagnosis in PMSMs Based on HO-VMD and Weighted Evidence Fusion of Current–Vibration Signals

**DOI:** 10.3390/s25154591

**Published:** 2025-07-24

**Authors:** Xianwu He, Xuhui Liu, Cheng Lin, Minjie Fu, Jiajin Wang, Jian Zhang

**Affiliations:** 1 Sanmen County Rural Water Affairs Center, Taizhou 317100, China; xianwu_he@163.com; 2 Standard & Quality Control Research Institute Ministry of Water Resources, Hangzhou 310012, China; xuhui_liu2011@163.com; 3 Sanmen County Water Engineering Quality and Safety Affairs Center, Taizhou 317100, China; cheng_lin2011@126.com; 4 College of Electrical Engineering, Zhejiang University, Hangzhou 310007, China; 3190100953@zju.edu.cn (M.F.); jian_zhang_zju@zju.edu.cn (J.Z.)

**Keywords:** Hippopotamus Optimization Variational Mode Decomposition, Teager–Kaiser Energy Operator, weighted modified Dempster–Shafer evidence theory, current and vibration signals, early bearing fault diagnosis, permanent magnet synchronous motor

## Abstract

To address the challenges posed by weak early fault signal features, strong noise interference, low diagnostic accuracy, poor reliability when using single information sources, and the limited availability of high-quality samples in practical applications for permanent magnet synchronous motor (PMSM) bearings, this paper proposes an early bearing fault diagnosis method based on Hippopotamus Optimization Variational Mode Decomposition (HO-VMD) and weighted evidence fusion of current–vibration signals. The HO algorithm is employed to optimize the parameters of VMD for adaptive modal decomposition of current and vibration signals, resulting in the generation of intrinsic mode functions (IMFs). These IMFs are then selected and reconstructed based on their kurtosis to suppress noise and harmonic interference. Subsequently, the reconstructed signals are demodulated using the Teager–Kaiser Energy Operator (TKEO), and both time-domain and energy spectrum features are extracted. The reliability of these features is utilized to adaptively weight the basic probability assignment (BPA) functions. Finally, a weighted modified Dempster–Shafer evidence theory (WMDST) is applied to fuse multi-source feature information, enabling an accurate assessment of the PMSM bearing health status. The experimental results demonstrate that the proposed method significantly enhances the signal-to-noise ratio (SNR) and enables precise diagnosis of early bearing faults even in scenarios with limited sample sizes.

## 1. Introduction

As the core power unit of industrial automation systems, PMSMs are widely employed across various industrial sectors due to their high efficiency and reliability. Statistics indicate that over 40% of motor failures are attributed to bearing faults [1], which directly affect the equipment performance and operational reliability. Therefore, timely and accurate early fault diagnosis of motor bearings is crucial for reducing the production downtime, lowering maintenance costs, and enhancing system reliability and economic efficiency.

Currently, vibration-based methods for bearing fault diagnosis are extensively studied and widely applied [2]. The stator current of PMSMs also contains substantial fault information; therefore, motor current signature analysis has emerged as an important diagnostic approach, as it is immune to environmental interference and does not require additional sensors [3]. In recent years, to address the challenges of weak early fault features and a low SNR, researchers have proposed novel methods such as deep learning, time–frequency analysis, and multi-scale decomposition, which have greatly improved the diagnostic sensitivity and accuracy [4,5]. Among these, deep learning and other artificial intelligence (AI) algorithms—owing to their strong feature extraction and pattern recognition capabilities—have become research hotspots in the field of bearing fault diagnosis. However, these methods typically rely heavily on large quantities of high-quality, accurately labeled training samples. The generalization and practical effectiveness of such models can be ensured only when the available data are sufficiently abundant and diverse. In real industrial environments, complex and variable signal interference and noise often limit both the availability and quality of training samples, making it difficult for deep learning models to effectively capture early fault features. This limitation may lead to reduced diagnostic accuracy and robustness. Furthermore, traditional diagnostic methods relying on a single signal source often suffer from inadequate information utilization and are highly susceptible to noise and harmonic interference under complex operating conditions, further restricting the accuracy and generalization capability of fault identification [6].

To address the challenges of weak early fault signatures and susceptibility to noise, signal decomposition and denoising are commonly employed techniques. Classical methods such as Empirical Mode Decomposition (EMD) [7] and Local Mean Decomposition (LMD) [8] can decompose non-stationary signals but often suffer from mode mixing and spurious frequency components. VMD [9] overcomes these limitations; however, its performance is highly dependent on the selection of the number of modes and the penalty parameter. Consequently, integrating VMD with parameter optimization algorithms [10] can significantly improve the decomposition performance and enhance the SNR of the extracted features.

For parameter optimization, traditional algorithms such as Particle Swarm Optimization (PSO) [11] and the Genetic Algorithm (GA) [12] are commonly used for optimizing VMD parameters. However, these algorithms often suffer from premature convergence, a tendency to get trapped in local optima, and a slow convergence speed. The recently developed HO algorithm integrates multiple strategies to more effectively escape local optima and provides a faster and more robust global search capability [13]. HO has demonstrated strong parameter optimization performance in neural networks such as Long Short-Term Memory (LSTM) and Backpropagation Neural Networks (BPNNs) [14,15]. Therefore, this paper applies HO to VMD parameter tuning to further enhance signal decomposition and feature extraction.

For early bearing fault signals, demodulation transforms are also effective in suppressing noise and improving the SNR. Traditional demodulation methods, such as the Hilbert transform [16], Park vector transform [17], and envelope spectrum analysis [18], can suppress fundamental frequency components in the current and enhance the SNR. However, these methods are insufficiently sensitive to early-stage and non-stationary signals. Therefore, this paper employs the Teager–Kaiser Energy Operator (TKEO) [19], which is highly sensitive to impulsive components, to process early bearing fault signals and effectively enhance early fault features.

Moreover, multi-source information fusion is widely adopted to improve the accuracy and robustness of fault diagnosis. In contrast to single-source approaches, multi-source fusion leverages information from vibrations, the current, the temperature, and other signals to enhance feature representation and reduce misclassifications caused by noise or weak signals, thereby improving the diagnostic reliability [20]. However, the existing methods, such as fuzzy set theory [21], neural networks [22], and traditional Dempster–Shafer evidence theory (TDST) [23], each have their limitations. For example, fuzzy set theory lacks a unified and rigorous scientific foundation, neural networks require large quantities of high-quality training data, and the TDST may fail when dealing with highly conflicting evidence. To address these challenges, this paper introduces a weighting mechanism to improve the Dempster–Shafer theory, thereby enhancing its adaptability and effectiveness in early bearing fault diagnosis.

To address the challenges of insufficient early fault samples, low diagnostic accuracy, poor reliability, and susceptibility to noise and harmonic interference in the early fault diagnosis of PMSM bearings, this paper proposes a novel method based on HO-VMD and weighted evidence fusion of current and vibration signals. In comparison with deep learning and other artificial intelligence approaches, the proposed method is grounded in clear physical principles, does not require large amounts of training data, and can be flexibly adjusted according to the engineering expertise. These advantages make it particularly suitable for small-sample diagnosis under complex operating conditions.

The main contributions of this work are as follows:

1. The proposed method adaptively determines the optimal VMD parameters using the HO algorithm, enabling efficient decomposition of early fault current and vibration signals. Signal reconstruction is performed based on kurtosis, effectively suppressing noise and harmonics.
2. The TKEO is employed for demodulation to enhance the fault features. The time-domain kurtosis and feature noise ratio (FNR) are extracted from the reconstructed signals to construct multi-source information. Weighted coefficients are assigned based on the feature reliability to adjust the BPA functions of the multi-source information.
3. A directed Softmax-based continuous similarity function is introduced to determine the BPA functions. On this basis, the WMDST is applied to achieve multi-source information fusion and early fault diagnosis.

The structure of this paper is as follows: Section 2 presents an overview of the primary fault signal analysis methods. Section 3 describes the experimental platform and the procedure for early bearing fault diagnosis. Section 4 evaluates the effectiveness, accuracy, and superiority of the proposed method through a series of comparative experiments. Section 5 discusses the results and provides an outlook for future research, while Section 6 concludes the study.

## 2. Fundamental Theories of Fault Signal Analysis

### 2.1. Feature Analysis of Fault Current and Vibration Signals

Most PMSM bearings are rolling bearings, consisting of an outer race, inner race, rolling elements, and a cage. Common faults include damage to the outer race, inner race, and rolling elements. During operation, the outer race typically remains stationary, while the inner race rotates with the rotor, making the outer race more susceptible to damage [24]. Notably, when faults occur in the inner race or rolling elements, the manifestation of fault features in both the vibration and current signals is similar to that of outer race faults, differing only in their characteristic frequencies and amplitudes. Therefore, the diagnostic process for inner race and rolling element faults follows the same procedure as for outer race faults [25]. For this reason, this study focuses on experimental analysis of outer race faults, which have the highest probability of occurrence, as the primary example. The characteristic frequency of ORFs is calculated as follows [26]:

(1) $$f_{o}=\frac{Z}{2}\left(1-\frac{d}{D_{c}}\cos\theta \right)f_{r}$$

where *f_o_* is the characteristic frequency of outer race faults (ORFs), *Z* is the number of rolling elements, *f_r_* is the rotational frequency, *d* is the diameter of a rolling element, *D_c_* is the pitch diameter of the bearing, and *θ* is the contact angle.

When a bearing fault occurs, the fault location generates periodic impacts, resulting in periodic variations in the air gap of the motor. This leads to periodic changes in the motor inductance, which ultimately affect the stator current signal and produce specific fault characteristic frequency components. Numerous studies have investigated and verified this characteristic frequency [27], and its calculation formula is as follows:

(2) $$f_{bf}=\left|f_{1}\pm pf_{o}\right|$$

where *f_bf_* is the ORF component in the stator current signal, *f*_1_ is the fundamental frequency of the stator current, and *p* = 1, 2, …

### 2.2. Hippopotamus Optimization Variational Mode Decomposition

VMD has been widely applied in fault diagnosis due to its strong noise immunity and ability to suppress mode mixing. However, its decomposition performance is highly dependent on the selection of the mode number and penalty factor, necessitating the use of appropriate optimization algorithms for parameter tuning [28]. The HO algorithm, inspired by the foraging behavior of hippopotamuses, achieves a balance between global and local searches, offering fast convergence and strong robustness. Therefore, this paper integrates HO with VMD to realize adaptive decomposition and denoising of fault current and vibration signals. The algorithmic procedure is as follows.

#### 2.2.1. Variational Mode Decomposition

The decomposition process of VMD essentially involves solving a variational problem and can be divided into the construction and solving of the variational problem. In order to decompose the original signal *x*(*t*) into several modal components, *u_k_*(*t*), with a limited bandwidth and to minimize the bandwidth of each mode, the following variational constrained problem is formulated:

(3) $$\left\{\begin{array}{l} \underset{\left\{u_{k}\right\},\left\{\omega_{k}\right\}}{\min}\left\{\sum_{k}{\left\|\partial_{t}\left[\left(\delta (t)+\frac{j}{\pi t}\right)*u_{k}\left(t\right)\right]e^{-j\omega_{k}t}\right\|}_{2}^{2}\right\}\\ s.t.\sum_{k=1}^{K}u_{k}\left(t)=x(t\right)\; \end{array}\right.\;$$

where *δ*(*t*) is the Dirac delta function, *j* is an imaginary unit, *ω_k_* is the center frequency of the *k*th mode function, and ‖•‖_2_ represents the *L*_2_ norm.

To solve the above variational problem, a penalty factor, *α*, and a Lagrange multiplier, *λ*(*t*), are introduced, resulting in the following augmented Lagrangian expression:

(4) $$\begin{array}{c} L(\{u_{k}\},\{\omega_{k}\},\lambda )=\alpha {\sum_{k}\left\|\partial_{t}\left[\left(\delta (t)+\frac{j}{\pi t}\right)*u_{k}(t)\right]e^{-j\omega_{k}t}\right\|}_{2}^{2}+{\left\|x(t)-\sum_{k}u_{k}(t)\right\|}_{2}^{2}\\ \;+\langle \lambda (t),x(t)-\sum_{k}u_{k}(t)\rangle \end{array}$$

By iteratively solving Equation (4) using the Alternating Direction Method of Multipliers (ADMM), the optimal solution to the variational problem can be obtained. The specific steps are as follows:

1. Initialize {$u_{k}^{1}$}, {$\omega_{k}^{1}$}, {$\lambda_{k}^{1}$}, and *n*.
2. Update *u_k_* according to the following formula:
  (5) $$u_{k}^{n+1}=\arg_{u_{k}}\min L\left(\{u_{i<k}^{n+1}\},\{u_{i\geq k}^{n}\},\{\omega_{i}^{n}\},\lambda^{n}\right)$$
3. Update *ω_k_* according to the following formula:
  (6) $$\omega_{k}^{n+1}=\arg_{\omega_{k}}\min L\left(\{u_{i}^{n+1}\},\{\omega_{i<k}^{n}\},\{\omega_{i\geq k}^{n}\},\lambda^{n}\right)$$
4. Update the Lagrange multiplier *λ* according to the following formula:
  (7) $$\lambda^{n+1}=\lambda^{n}+\tau \left(x(t)-\sum_{k}u_{k}^{n+1}(t)\right)$$
  where *τ* is the step size factor.
5. Repeat steps 2–4 until the stopping condition in Equation (8) is satisfied. Then, terminate the iteration to obtain *K* IMFs.
  (8) $$\sum_{k}\frac{{\left\|u_{k}^{n+1}-u_{k}^{n}\right\|}_{2}^{2}}{{\left\|u_{k}^{n}\right\|}_{2}^{2}}<\varepsilon$$

#### 2.2.2. Hippopotamus Optimization Algorithm

The number of modes, *K*, and the penalty factor, *α*, in VMD need to be optimally selected. The optimization procedure of the HO algorithm is as follows:

1. Define the optimization parameter set (*K*, *α*), and set the optimization boundaries as *K*_min_ ≤ *K* ≤ *K*_max_, *α*_min_ ≤ *α* ≤ *α*_max_. Since the optimization steps for *K* and *α* are the same, the following takes *K* as an example for illustration.
2. Population initialization: Multiple solutions are randomly generated to construct the initial population.
  (9) $$K_{i}=K_{\min}+\mathrm{round}\left[rand(0,1)\times (K_{\max}-K_{\min})\right]$$
3. Construct the fitness function: To more accurately identify early bearing faults, this paper combines the envelope entropy and kurtosis—both sensitive to impulsive signal components—to construct the following fitness function:
  (10) $$E_{k}(n)=-\sum_{j=1}^{N}\frac{e_{k}(n)}{\sum e_{k}(n)}\mathrm{lg}\frac{e_{k}(n)}{\sum e_{k}(n)}$$
  (11) $$q_{k}=\frac{1}{N}\sum_{n=1}^{N}{\left(u_{k}(n)-\mu_{k}\right)}^{4}/{\left[\frac{1}{N}\sum_{n=1}^{N}{\left(u_{k}(n)-\mu_{k}\right)}^{2}\right]}^{2}$$
  (12) $$\left\{\begin{array}{l} C_{i}=E_{k}(n)+\frac{1}{\left|q_{k}-3\right|}\;\;\;\;\mathrm{Vibration}\\ C_{i}=E_{k}(n)+\frac{1}{\left|q_{k}-2.5\right|}\;\mathrm{Current} \end{array}\right.$$
  where *C_i_* is the optimal fitness value of the *i*th set of optimization parameters. The difference between the current and vibration signals lies in the kurtosis under healthy conditions. *N* is the number of sampling points of the modal function *u_k_*(*n*); *e_k_*(*n*) is the Hilbert envelope of *u_k_*(*n*); *q_k_* is the kurtosis of *u_k_*(*n*); and *μ_k_* is the mean value of *u_k_*(*n*).
4. Global search phase: The foraging behavior of hippopotamuses is simulated to explore the entire solution space and achieve global updates. The mathematical model incorporates random perturbations to avoid falling into local optima.
  (13) $$K_{i}^{v+1}=K_{i}^{v}+z_{1}\cdot (K_{\mathrm{best}}^{v}-K_{i}^{v})+z_{2}\cdot (K_{\mathrm{rand}}^{v}-K_{i}^{v})$$
  where $K_{\mathrm{best}}^{v}$ is the position of the best individual in the current population, $K_{\mathrm{rand}}^{v}$ represents the position of a randomly selected individual in the current population, *v* is the iteration number, and *z*_1_ and *z*_2_ are random numbers in the range [0, 1].
5. Local exploitation phase: This phase simulates the concentrated foraging behavior of hippopotamuses in high-quality areas, performing fine searches in regions with high fitness to improve the quality of the solution.
  (14) $$K_{i}^{v+1}=K_{i}^{v}+z_{3}\cdot (K_{\mathrm{best}}^{v}-K_{\mathrm{rand}}^{v})$$
  where *z*_3_ is a random number in the range [0, 1].
6. Dynamic switching of exploration strategies: An exploration probability, *h*, is defined. When the random number *s* < *h*, the global exploration strategy is selected; when *s* > *h*, the local exploitation strategy is chosen. In general optimization problems, exploration is emphasized in the early stages while exploitation is prioritized in the later stages. Therefore, *h* is defined as follows:
  (15) $$h=h_{0}\times (1-\frac{v}{V})$$
  where *h*_0_ is the initial exploration probability, and *V* is the maximum number of iterations.

The iteration terminates when the maximum number of iterations is reached or the optimal solution with the minimum *C_i_* is found.

**Figure 1:**
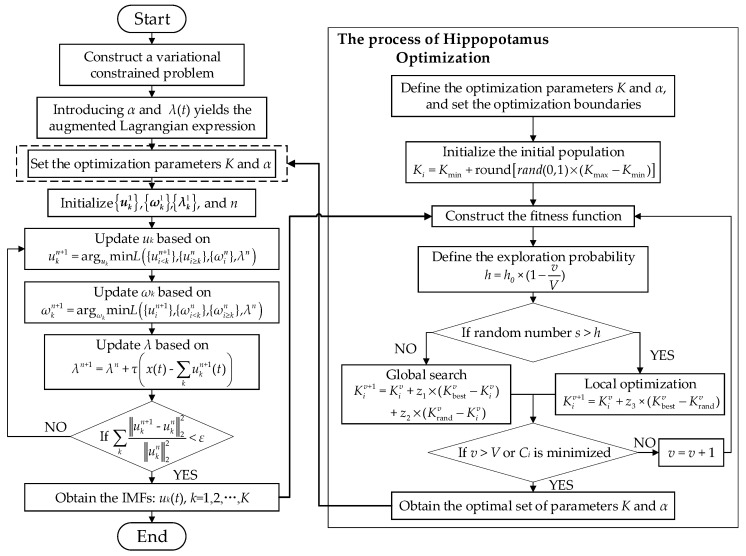
HO-VMD process.

The specific steps of HO-VMD are illustrated in Figure 1.

### 2.3. Teager–Kaiser Energy Operator

The TKEO is an effective nonlinear energy analysis tool suitable for enhancing the instantaneous features of signals and extracting modulation information. For a discrete signal, *x*(*n*), the TKEO is defined as

(16) $$\Psi [x(n)]=x{\left(n\right)}^{2}-x(n-1)\cdot x(n+1)$$

For a frequency- and amplitude-modulated fault signal, *x*(*n*) = *A*(*n*)cos(*φ*(*n*)), the output of the TKEO after demodulation can be approximated as

(17) $$\Psi [x(n)]\approx A{\left(n\right)}^{2}\omega {\left(n\right)}^{2}$$

where *A*(*n*) is the instantaneous amplitude, *φ*(*n*) is the instantaneous phase, and *ω*(*n*) is the instantaneous frequency.

Compared with traditional demodulation methods, the TKEO can effectively detect instantaneous frequency components [29] and enhance impulsive early fault features, thereby facilitating the extraction of weak fault information.

### 2.4. Weighted Modified Dempster–Shafer Evidence Theory

The DST offers significant advantages in fusing uncertain multi-source information. To improve its adaptability in handling highly conflicting evidence, this paper introduces a weighting scheme and conflict management mechanism to achieve effective fusion of multi-source feature information.

Let the frame of discernment be a finite set, Θ, and its power set be 2^Θ^, which contains all possible subsets of Θ. A function, *m*: 2^Θ^ ⊆ [0, 1], is defined such that

(18) $$\left\{\begin{array}{l} m(\emptyset )=0\\ \sum_{A\subseteq 2^{\Theta }}m(A)=1 \end{array}\right.$$

where *m*(*A*) is the BPA function representing the degree of belief assigned to subset *A*, and ∅ denotes the empty set.

In this study, a directed Softmax-based continuous similarity function is employed to construct the BPA functions between the feature values and each standard state. For a given feature parameter, *w*, let the standard values be *W* = {*w*_f_, *w*_u_, *w*_h_}, corresponding to the fault, uncertain, and healthy states, respectively. The probability assignments are given by

(19) $$\left\{\begin{array}{l} m_{f}=\exp[\xi (w-w_{f})]\\ m_{u}=\exp[-\xi \left|w-w_{u}\right|]\\ m_{h}=\exp[-\xi (w-w_{h})] \end{array}\right.$$

where *ξ* is a tuning parameter that can be adjusted according to different standard value intervals. The BPA functions are normalized such that ∑*m_i_* = 1.

Suppose the BPA functions of two evidence sources, *E*_1_ and *E*_2_, are *m*_1_ and *m*_2_, respectively. The TDST is given by

(20) $$\left\{\begin{array}{l} m_{12}(C_{l})=m_{1}(A_{i})⊕m_{2}(B_{g})=\frac{1}{1-T}\sum_{A_{i}\cap B_{g}=C_{l}}m_{1}(A_{i})m_{2}(B_{g})\;C_{l}\neq \emptyset \\ m_{12}(C_{l})=0C_{l}=\emptyset \end{array}\right.$$

(21) $$T=\sum_{A_{i}\cap B_{g}=\emptyset }m_{1}(A_{i})m_{2}(B_{g})$$

where *C_l_* denotes the common subset of *A_i_* and *B_g_*, and *T* is the conflict coefficient between *m*_1_ and *m*_2_. A larger value of *T* indicates greater conflict; when *T* → 1 (i.e., highly conflicting evidence), the fusion result may become unreliable [30]. Therefore, it is necessary to modify the evidence fusion algorithm.

To improve the handling of highly conflicting evidence, weighted sum-of-squares conflict allocation is employed. The modified evidence fusion formula is given by

(22) $$\left\{\begin{array}{l} m_{12}(C_{l})=\sum_{A_{i}\cap B_{g}=C_{l}}m_{1}(A_{i})m_{2}(B_{g})+a_{l}T\;C_{l}\neq \emptyset \\ m_{12}(C_{l})=0\;C_{l}=\emptyset \end{array}\right.$$

where *a_l_* is the conflict allocation coefficient, defined as

(23) $$a_{l}=\frac{\sum_{A_{i}\cap B_{g}=C_{l}}(m_{1}^{2}(A_{i})+m_{2}^{2}(B_{g}))}{\sum (m_{1}^{2}(A_{i})+m_{2}^{2}(B_{g}))}$$

Furthermore, to reflect the actual reliability of each evidence source, weighting coefficients are introduced. Suppose the weighting coefficients of *E*_1_ and *E*_2_ are *γ*_1_ and *γ*_2_, respectively, where *γ*_1_ + *γ*_2_ = 1. If *E*_1_ is more reliable, then *γ*_1_ is assigned a larger value. The average degree of support provided by *N* evidence sources for a given subset is defined as

(24) $$\bar{m}=\sum_{i=1}^{N}\gamma_{i}m_{i}$$

The weighted BPA functions are thus given by

(25) $$\left\{\begin{array}{l} m_{1}\prime =\gamma_{1}m_{1}+(1-\gamma_{1})\bar{m}\\ m_{2}\prime =\gamma_{2}m_{2}+(1-\gamma_{2})\bar{m} \end{array}\right.$$

After calculation, normalization is performed so that $\sum m_{i}\prime$ = 1.

Finally, the WMDST fusion formula is given by

(26) $$\left\{\begin{array}{l} m_{12}\prime (C_{l})=\sum_{A_{i}\cap B_{g}=C_{l}}m_{1}\prime (A_{i})m_{2}\prime (B_{g})+a_{l}\prime T^{\prime }\;C_{l}\neq \emptyset \\ m_{12}\prime (C_{l})=0\;C_{l}=\emptyset \end{array}\right.$$

## 3. Experimental Platform and Early Bearing Fault Diagnosis Procedure

### 3.1. Experimental Platform

**Figure 2:**
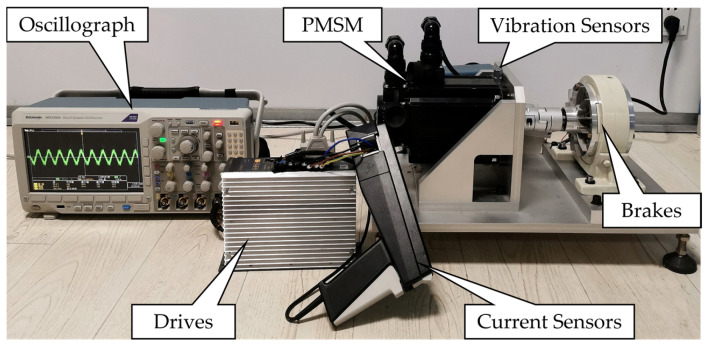
Experimental motor platform.

As shown in Figure 2, the experimental platform consisted of three subsystems: a power supply system, a drive system, and a data acquisition system. The motor used in the experiments was a surface-mounted permanent magnet synchronous motor with eight poles and thirty-six slots rated at 1.5 kW. A magnetic powder brake was used to apply and control the load on the motor. The data acquisition system included a BSZ800D-16 vibration collector, a TCP303 current probe, and an oscilloscope, enabling synchronous acquisition of both vibration and stator current signals.

**Figure 3:**
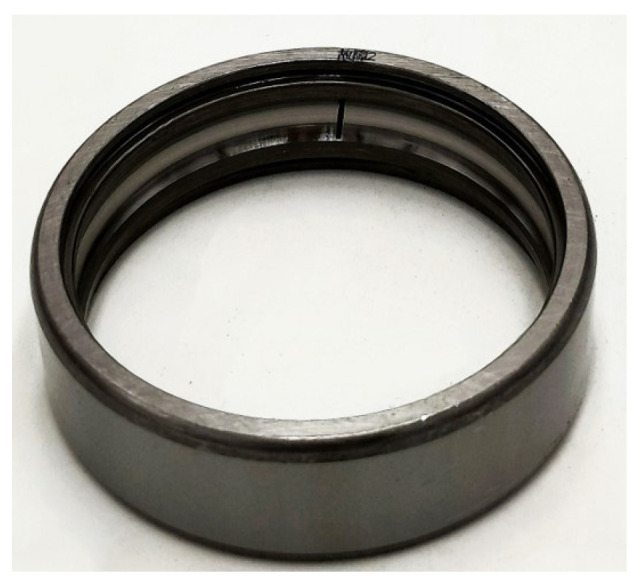
Bearing with ORF.

The bearing fault was simulated using a notch machining method. As CWRU used a notch width of 0.007 inches (0.1778 mm) to simulate the smallest bearing fault they examined [31,32,33], this study referred to their experimental scheme and machined a 0.2 mm wide notch on the outer race of a 6205-type bearing to simulate an early-stage fault condition. The faulty bearings are shown in Figure 3, and the parameters are listed in Table 1. During the experiment, the motor ran steadily at 1500 rpm, and both the vibration and current signals were sampled at a frequency of 25 kHz.

Regarding the calculated parameters, the fundamental frequency of the current, *f_s_*, was 100 Hz, the rotational frequency, *f_r_*, was 25 Hz, and the characteristic frequency of the outer race faults, *f_o_*, was 89 Hz.

### 3.2. Early Bearing Fault Diagnosis Procedure Based on Multi-Source Feature Information and WMDST

The proposed early bearing fault diagnosis method based on HO-VMD and weighted evidence fusion of current–vibration signals fully leverages the adaptive parameter optimization capabilities of HO-VMD for denoising and decomposing bearing fault signals. Multi-source features are then extracted using the TKEO, and information fusion as well as fault state diagnosis are achieved using the WMDST. The specific diagnostic procedure is as follows:

1. Data Acquisition: On the experimental platform, vibration and stator current signals corresponding to an early ORF in the bearing are synchronously collected using a vibration sensor and a current probe.
2. Signal Decomposition: The sampled signals are decomposed using HO-VMD, in which both the number of modes *K* and the penalty factor *α* are adaptively optimized through HO. The parameters are set as follows: *K* ranges from 2 to 10 with a step size of 1; *α* ranges from 0 to 2000 with a step size of 100; the population size is 10; the initial exploration probability *h*_0_ is 0.8; and the maximum number of iterations, *V*, is 20. The optimization ranges and step sizes were chosen based on references [34,35] and can adequately cope with the level of signal complexity encountered in this experiment.
3. Component Selection and Signal Reconstruction: The kurtosis value for each IMF is computed according to Equation (11). For signal reconstruction and denoising, IMFs with a kurtosis greater than 2.5 are selected from the current signal, while those with a kurtosis greater than 3 are selected from the vibration signal. The kurtosis threshold for the vibration signals is determined with reference to the kurtosis of a normal distribution signal. In contrast, the threshold for the current signals is set lower, since current signals typically contain fewer impulsive components than vibration signals.
4. Feature Extraction: After reconstructing the signals, their kurtosis is calculated, and the signals are subsequently demodulated using the TKEO. To further extract frequency-domain features from the energy spectrum, the total harmonic distortion [36] has been proposed as a quantitative measure of the severity of the current distortion induced by bearing faults. Building upon this concept, the fault-excited harmonic distortion [37] has been introduced to suppress interference from non-fault-related harmonic components, thereby enabling a more accurate estimation of the harmonic distortion specifically attributable to bearing faults. In this study, to extend the application of this feature indicator to vibration signals affected by faults, the fundamental component of the current is replaced with the average noise amplitude. Accordingly, we propose the use of the FNR to quantitatively capture the frequency-domain changes induced by bearing faults. The FNR is calculated as follows:
  (27) $$\mathrm{FNR}=\sqrt{A_{f_{1}}^{2}+A_{f_{2}}^{2}+\ldots +A_{fp}^{2}}/A_{fn}$$
  where *A_fp_* is the energy amplitude of the *p*th-order bearing fault characteristic component. Since the amplitude of the fault components decreases with an increasing order, only the first two orders are considered in the calculation. *A_fn_* is the average noise amplitude of the reconstructed signal’s TKEO energy spectrum, excluding the DC component. Meanwhile, as shown by the previously obtained experimental results in Table 2, the average noise amplitude increases substantially as the calculation frequency band widens. This is attributable to the presence of numerous harmonics in the high-frequency band that are unrelated to bearing faults, such as slot harmonics and inverter switching harmonics. The experimental results were obtained by averaging the results for 20 samples, each exhibiting clearly identifiable fault features. Because the amplitudes of the fault characteristic components in the high-frequency band are relatively small and not considered in this study, the noise calculation is restricted to the 0–300 Hz frequency range. This approach better highlights the characteristics of bearing faults while effectively suppressing interference from high-frequency harmonics.
5. BPA Function Calculation: The time-domain and frequency-domain BPA functions for the reconstructed current and vibration signals are calculated using the Softmax similarity function. To meet the requirements of early bearing fault diagnosis and based on the results of prior experimental tests, the standard values are set as shown in Table 3. Specifically, the standard values for healthy and faulty conditions are obtained by averaging the values of 20 samples with clearly defined healthy or faulty states for each condition, while the standard value for the uncertain state is defined as the average of the healthy and faulty standard values. The parameters are set as *ξ*_VF_ = 1, *ξ*_VK_ = 1, *ξ*_CF_ = 4, and *ξ*_CK_ = 50. These adjustment parameters are determined according to the range of the standard values to ensure that the calculated BPA function values more accurately reflected the actual conditions.
6. Weighted Coefficient Assignment and Feature Information Fusion: Weighting coefficients are assigned based on the reliability of the information sources. Based on the differences in the standard values of faulty and healthy signals presented in Table 3, the FNR is found to be more sensitive to early bearing faults. Therefore, the weighting coefficient for the kurtosis-based feature is set to 0.3, while that for the FNR is set to 0.7. For early bearing faults, the fault features in current signals are often attenuated during transmission, whereas vibration signals can more accurately and clearly reflect the fault characteristics. Although the standard FNR value for the faulty current signal in Table 3 is relatively high, this is attributed to significant fluctuations in the FNR of current signals and does not imply higher sensitivity to early faults—this is further supported by the differences in the standard kurtosis values. Consequently, the weighting coefficient for current signals is set to 0.35, and for vibration signals, it is set to 0.65. The BPA functions are adjusted according to the assigned weights and subsequently fused using the WMDST through second-order weighted fusion.
7. State Determination: After the information is weighted and fused, the status of the bearing is determined according to the decision rules. Assume that the weighted fusion probability assignment function is *m* = {$m_{f}^{’}$, $m_{u}^{’}$, $m_{h}^{’}$}. The bearing is considered to be faulty if the following conditions are met: (1) $m_{f}^{’}>m_{u}^{’}$ and $m_{f}^{’}>m_{h}^{’}$, and (2) $m_{f}^{’}-m_{u}^{’}>\eta$ and $m_{f}^{’}-m_{h}^{’}>\eta$, where *η* is the decision threshold, which can be adjusted according to the required diagnostic sensitivity. In this study, to enable early fault diagnosis of bearings, *η* is set to 0.35 based on experimental testing. The selection of this threshold has a significant impact on the accuracy of early fault diagnosis for bearing outer race faults. Fault diagnosis was performed on 50 early fault samples and 50 healthy samples, with the results shown in Table 4. If the threshold is set too high, fault samples may be misclassified as “healthy”; conversely, if it is set too low, healthy samples may be misclassified as “faulty”. Considering the overall diagnostic accuracy for early bearing outer race faults, *η* is set to 0.35.

**Figure 4:**
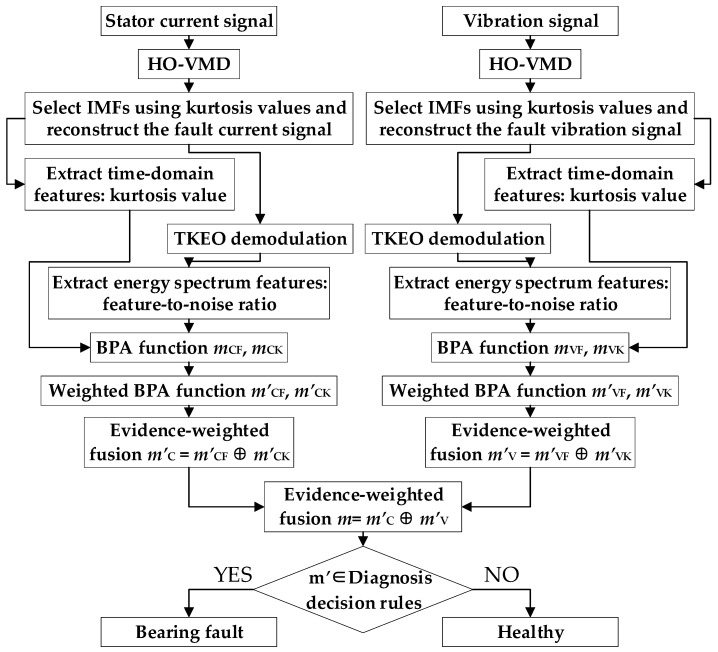
Early bearing fault diagnosis process using multi-source feature information and WMDST.

The overall process is illustrated in Figure 4.

## 4. Experimental Results and Analysis

**Figure 5:**
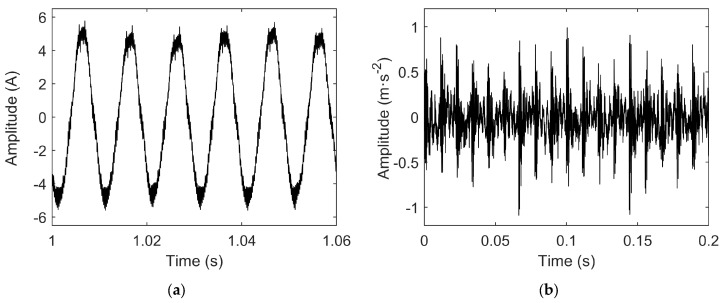
(**a**) Time-domain waveform of fault current signal; (**b**) time-domain waveform of fault vibration signal.

To validate the effectiveness of the proposed early bearing fault diagnosis method, current and vibration signals were sampled under no-load experimental conditions for a motor with an outer race bearing fault. The time-domain waveforms of the sampled fault signals are shown in Figure 5.

### 4.1. Decomposition and Reconstruction of Fault Current and Vibration Signals

**Figure 6:**
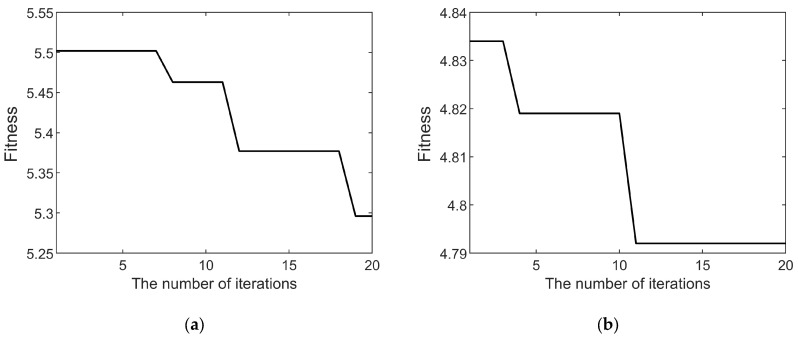
(**a**) PSO algorithm fitness curve of decomposition parameters for fault current signal; (**b**) PSO algorithm fitness curve of decomposition parameters for fault vibration signal.

**Figure 7:**
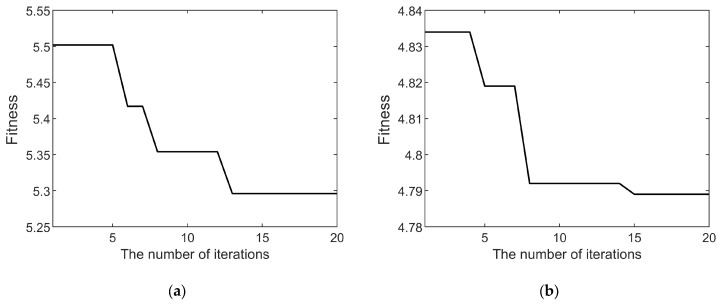
(**a**) HO algorithm fitness curve of decomposition parameters for fault current signal; (**b**) HO algorithm fitness curve of decomposition parameters for fault vibration signal.

To demonstrate the global search capability and rapid convergence of the HO algorithm when used for optimizing VMD decomposition parameters, both the widely used PSO algorithm and the HO algorithm were employed to iteratively optimize the parameter combinations (*K*, *α*) for the decomposition of the fault current and vibration signals. The variation in the fitness values during the iterative processes of the PSO and HO algorithms are illustrated in Figure 6 and Figure 7, respectively. As the number of iterations increases, the fitness values gradually decrease and eventually converge. A constant fitness value indicates that the optimization algorithm has identified the optimal decomposition parameters for the corresponding signal.

A comparison between Figure 6 and Figure 7 clearly demonstrates that the HO algorithm achieves a significantly faster convergence rate than the PSO algorithm in VMD parameter optimization. Furthermore, the final converged fitness value for the vibration signal is 4.792 with the PSO algorithm and 4.789 with the HO algorithm, indicating that the HO algorithm achieves a superior decomposition effect for the fault vibration signal. This result suggests that the PSO algorithm becomes trapped in a local optimum and fails to identify the global optimal decomposition parameters, whereas the HO algorithm successfully obtains the global optimum. Specifically, with the HO algorithm, the fitness value for the fault current signal converges to 5.296, and that for the vibration signal converges to 4.789. The corresponding optimal parameters obtained by the HO algorithm are *K* = 6 and *α* = 1200 for the current signal and *K* = 7 and *α* = 800 for the vibration signal.

**Figure 8:**
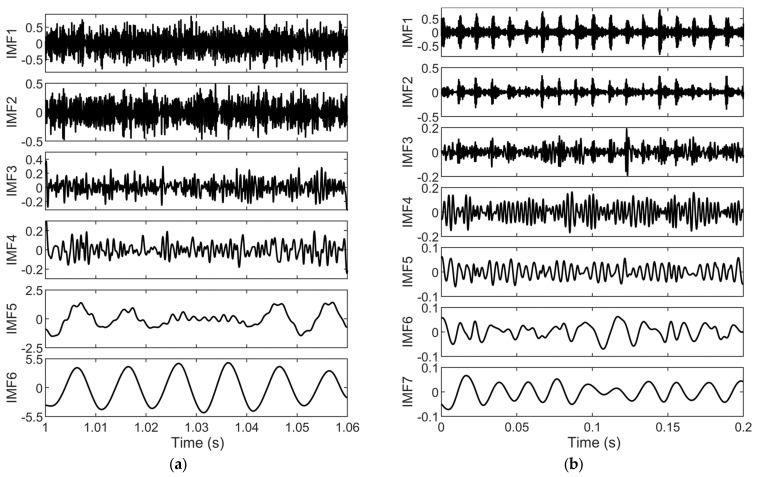
(**a**) Decomposition results for fault current signal; (**b**) decomposition results for fault vibration signal.

As shown in Figure 8, both the fault current and vibration signals are decomposed through VMD into several IMFs covering different frequency bands. For the fault current signal, the decomposed IMF6 has a period of approximately 0.01 s and an amplitude similar to that of the original signal, indicating that this component primarily reflects the fundamental frequency component of the fault current. The other IMFs contain information related to the inherent eccentric frequencies, bearing ORF characteristics, and environmental noise. For the vibration signal, the decomposition results show that the first three IMFs have fluctuation periods similar to those of the original vibration signal and mainly capture its principal frequency features, while the remaining components primarily reflect the noise and harmonic content.

The kurtosis of each IMF obtained from the decomposition of the fault current and vibration signals is calculated, as shown in Table 5. The analysis reveals that the kurtosis of the IMFs from the current signal is generally lower than that of the vibration signal. Moreover, the first two IMFs of the vibration signal exhibit a significantly higher kurtosis than the baseline value of 3 for normally distributed signals, indicating that the vibration signal contains more impulsive components and is more sensitive to bearing faults. Therefore, vibration features are assigned higher weights than current features in the subsequent information fusion process.

**Figure 9:**
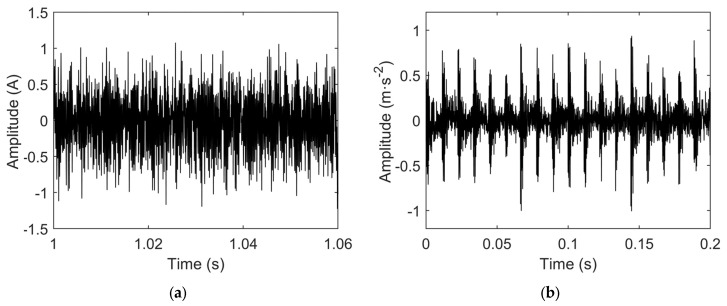
(**a**) Time-domain waveform of reconstructed fault current signal; (**b**) time-domain waveform of reconstructed fault vibration signal.

To further suppress the impact of environmental noise and the interference of the fundamental frequency of the current on the characteristic fault frequency of the bearing and to highlight key fault features for early fault diagnosis, a kurtosis-based selection method is adopted: IMFs with a kurtosis greater than 2.5 are selected from the current signal while those with a kurtosis greater than 3 are selected from the vibration signal for signal reconstruction. The reconstruction results are shown in Figure 9.

As shown in Figure 9, the time-domain waveform of the reconstructed current signal differs markedly from that of the original fault current signal. This difference arises mainly because the IMF6 component, which contains the fundamental frequency, is excluded during the IMF selection process. This exclusion effectively suppresses the effect of interference from the fundamental frequency on the fault characteristic frequency. Conversely, the vibration signal is more sensitive to bearing faults, with its original waveform clearly influenced by fault-induced fluctuations. After kurtosis-based selection, the reconstructed vibration signal effectively retains the characteristic frequency components associated with the bearing fault. Meanwhile, the environmental noise is significantly reduced. Consequently, the time-domain waveform of the reconstructed vibration signal closely resembles that of the original signal.

### 4.2. Multi-Source Feature Information Fusion and Early Bearing Fault Diagnosis

**Figure 10:**
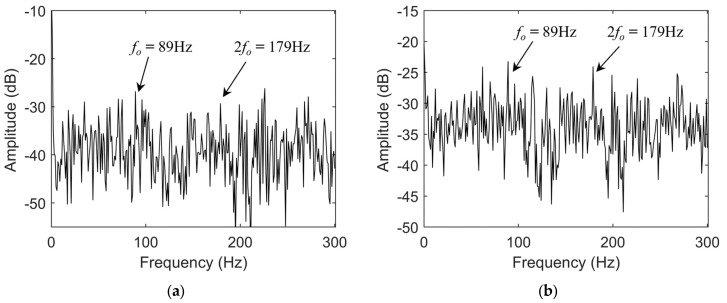
(**a**) Hilbert envelope spectrum of reconstructed fault current signal; (**b**) Hilbert envelope spectrum of reconstructed fault vibration signal.

Since the time-domain waveform of the fault signal contains limited fault feature information, it is difficult to accurately determine the existence of bearing faults using only time-domain analysis, whether from current or vibration signals. Therefore, spectral analysis is necessary to extract key characteristic components. As a commonly used demodulation method, the Hilbert transform can effectively highlight the fault features in the spectrum. Figure 10 presents the Hilbert envelope spectra of the reconstructed current and vibration signals, where the arrows indicate the characteristic fault frequency of the bearing outer race and its second harmonic. The Hilbert transform demodulates the modulated fault components in the current signal, so the envelope spectrum exhibits the fault characteristic frequency.

**Figure 11:**
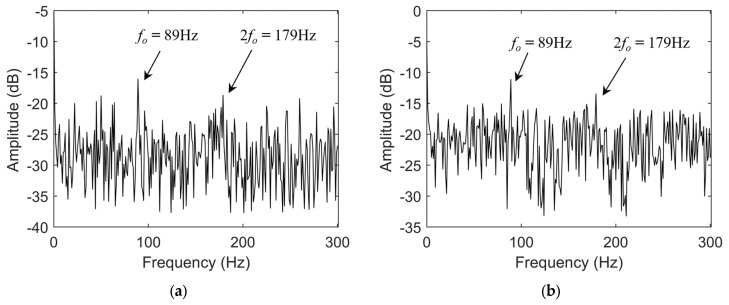
(**a**) TKEO energy spectrum of reconstructed fault current signal; (**b**) TKEO energy spectrum of reconstructed fault vibration signal.

As shown in the figure, the amplitude of the fault characteristic frequency in the envelope spectrum of the reconstructed current signal is relatively low and susceptible to environmental noise, making it difficult to accurately assess the bearing’s health status. In contrast, the fault characteristic frequency in the envelope spectrum of the reconstructed vibration signal is more prominent; however, its amplitude remains limited, making it challenging to achieve highly sensitive identification of early bearing faults. To further enhance the instantaneous characteristic frequencies in the fault signals, this study employs the TKEO, which is sensitive to weak impulsive components, to demodulate the reconstructed signals. Figure 11 shows the energy spectra of the fault signals processed using the TKEO.

Compared with the Hilbert envelope spectrum, the overall amplitude of the TKEO energy spectrum is noticeably increased, which is attributed to the energy enhancement characteristic of the demodulation method. In the TKEO energy spectrum of the reconstructed current signal, the amplitude of the fault characteristic frequency is clearly higher than that of the environmental noise, which facilitates accurate diagnosis of early bearing faults. Similarly, the TKEO energy spectrum of the vibration signal exhibits even more prominent fault feature amplitudes, further validating the effectiveness of the TKEO energy spectrum in early bearing fault diagnosis.

To demonstrate the superiority of the TKEO energy spectrum in extracting frequency-domain features from bearing fault signals, this paper calculates the FNR characteristic index for both the fault current and vibration signals in the Hilbert envelope spectrum and the TKEO energy spectrum, respectively, according to Equation (27). The calculation results are presented in Table 6. It can be observed from Table 6 that the FNR of the TKEO energy spectrum is significantly higher than that of the Hilbert envelope spectrum, demonstrating that the TKEO energy spectrum more prominently reveals fault characteristics and is advantageous for early bearing fault feature extraction.

It should be noted that although the TKEO energy spectrum can effectively enhance the fault features and thereby improve the fault identification accuracy, relying solely on the frequency amplitude exceeding the noise levels or using a single FNR value as the basis for fault diagnosis still has certain limitations. This is particularly the case in environments with significant noise interference or when the fault features are inherently weak, as the diagnostic results obtained from a single-signal source often lack stability and are highly dependent on expert experience. To further improve the accuracy of early bearing fault diagnosis, this paper proposes a diagnostic method based on multi-source feature information fusion. Numerous studies have shown that the time-domain kurtosis is an effective feature indicator for distinguishing bearing states; however, relying exclusively on time-domain features makes it challenging to achieve high diagnostic accuracy. In light of this, the proposed method selects the FNR as the frequency-domain feature indicator and kurtosis as the time-domain feature indicator, fusing information from both the fault current and vibration signals. Based on the DST, multi-source information fusion is implemented to enable accurate identification of bearing states.

To further enhance the diagnostic robustness and accuracy, weighting coefficients are assigned to each feature information source based on their prior reliability. The extracted kurtosis and FNR features of both the current and vibration signals are then fused using the WMDST, which effectively avoids the failure of the TDST in highly conflicting cases. The calculated fault signal feature values are as follows: the current kurtosis *q*_c_ = 2.664, the current feature noise ratio FNR_c_ = 3.911; the vibration kurtosis *q*_v_ = 4.679; and the vibration feature noise ratio FNR_v_ = 3.446. Using Equation (19) and the standard feature values in Table 3, the BPA functions are computed, as shown in Table 7. The results indicate that, in the case of early and minor bearing faults, accurate fault diagnosis cannot be achieved by relying solely on the fault features of the current signal.

The calculated BPA functions are fused using both the TDST and the WMDST proposed in this paper. The fusion results show that using the TDST yields *m*_T_ = {0.157,0.842,0.001}, while using the WMDST yields *m*_W_ = {0.646,0.353,0.001}. These results indicate that, when there is a conflict among multi-source information, the TDST is significantly influenced by the evidence source *m*_CK_, which alone classifies the experimental bearing as having an “uncertain” state. As a result, the TDST ultimately diagnoses the bearing state as “uncertain”, which does not correspond to its actual state.

In contrast, the WMDST, through the use of weight allocation and conflict management mechanisms, provides a fusion result that accurately identifies the experimental bearing as being in the “fault” state, consistent with its real condition. However, validation experiments show that relying solely on the numerical values of the fused probability assignment function may still not guarantee completely accurate identification of the actual bearing state. To further improve the accuracy and reliability of the diagnosis, a second decision rule is introduced in this paper: $m_{f}^{’}-m_{u}^{’}>\eta$ and $m_{f}^{’}-m_{h}^{’}>\eta$, where *η* is set to 0.35 based on multiple experimental trials. Using this rule, when the BPA functions are fused using the WMDST without prior weighting adjustment, the results still fail to accurately identify the bearing as being in the “fault” category.

Since different fault feature information sources exhibit varying sensitivities to bearing faults, applying uniform weighting for evidence fusion is inappropriate. Such an approach can amplify the influence of conflicting information during fusion, resulting in reduced reliability and potentially causing misclassification of the actual bearing state. Therefore, in this study, weighting coefficients are assigned to each feature information source according to its prior reliability. The BPA functions presented in Table 7 are adjusted based on Equations (24) and (25), and the adjusted results are summarized in Table 8. Because the current time-domain features are less sensitive to bearing faults, they are assigned lower weighting coefficients. In contrast, information sources with higher reliability exert a stronger influence on the fusion results. After weighting adjustment, the BPA functions more accurately represent the actual bearing condition.

Subsequently, both the TDST and the WMDST are applied to fuse the adjusted BPA functions. The results show that using the TDST yields $m_{T}^{’}$ = {0.933,0.038,0.029}, while using the WMDST yields $m_{W}^{’}$ = {0.957,0.043,0.000}. It can be seen that after weighting adjustment, both evidence fusion methods can reliably and accurately identify early bearing faults, primarily because the conflicts among the feature information sources have been effectively reduced. A further comparison reveals that, compared with the TDST, the WMDST tends to more strongly indicate the presence of a fault, demonstrating higher diagnostic accuracy for early bearing fault identification.

### 4.3. Comparative Ablation Experiments on Early Bearing ORF Diagnosis

To further validate the effectiveness and accuracy of the proposed HO-VMD and current–vibration signal weighted evidence fusion method for early bearing fault diagnosis and to highlight its advantages over AI algorithms under limited sample conditions, this study conducted comparative ablation experiments. Specifically, 50 sets of faulty and 50 sets of healthy current–vibration data, acquired via synchronous sampling, were employed as experimental samples. For the AI algorithms, all 100 experimental samples were used for model testing, while an additional 25 sets each of the faulty and healthy samples were used for model training. The control groups included a method considering the vibration signal only, a method considering the current signal only, the TDST method, WaveCAResNet [4], AlexNet [38], and ConvNeXt-T [39].

Experiments using a single information source were designed to verify the advantages of the multi-source information fusion method in early bearing fault diagnosis. In these experiments, the reliability weights of the kurtosis and FNR indicators were kept consistent with those used in the multi-source information fusion experiments. The TDST method was included to demonstrate the superiority of the proposed WMDST method in managing conflicting information. WaveCAResNet represented recent advancements in multi-source information fusion diagnosis using AI algorithms. AlexNet was used to illustrate the diagnostic performance of lightweight convolutional neural networks, while ConvNeXt-T represented diagnostic models based on the Transformer architecture. The accuracy, precision, and recall of these seven bearing fault diagnosis methods were compared, and the calculation formulas were as follows:

(28) $$Accuracy=\frac{TP+TN}{TP+TN+FP+FN}$$

(29) $$Precision=\frac{TP}{TP+FP}$$

(30) $$Recall=\frac{TP}{TP+FN}$$

where *TP* denotes the number of samples where the true category was positive and correctly predicted by the model, *TN* denotes the number of samples where the true category was negative and correctly predicted by the model, *FP* denotes the number of samples where the true category was negative but incorrectly predicted by the model to be positive, and *FN* denotes the number of samples where the true category was positive but incorrectly predicted by the model to be negative. The experimental results are shown in Table 9.

Based on the comparative experimental results presented in Table 9, it is evident that using different diagnostic methods resulted in significant variations in key performance metrics, including the accuracy, precision, and recall. The diagnostic approach proposed in this paper consistently demonstrated outstanding performance across all three metrics, achieving an accuracy of 99.0%, a precision of 98.04%, and a recall rate of 100.0%. These results clearly underscore the advantages of WMDST-based multi-source information fusion in early bearing fault diagnosis.

In contrast, when weighted fusion was only applied to a single signal source, all the evaluation metrics decreased to varying extents. Notably, the method relying solely on the current signals exhibited the lowest performance, with the accuracy and precision decreasing to 76.0% and 74.07%, respectively. This decline was primarily due to the significant attenuation of the fault features in current signals during transmission, making early bearing faults more challenging to detect.

Although TDST-based multi-source information fusion was slightly less effective than that based on the WMDST, its overall accuracy remained high. This further demonstrates the effectiveness of the proposed method in resolving conflicting information and optimally allocating fusion weights.

For AI algorithms such as WaveCAResNet, AlexNet, and ConvNeXt-T, all three evaluation metrics remained below 90%, which was notably inferior to the results achieved using the method proposed in this study. This disparity was primarily due to the limited number of training samples in this experiment, which constrained the generalization ability and diagnostic accuracy of the AI-based methods. These findings indicate that, under small-sample conditions, deep learning and other AI approaches exhibit inherent limitations in early bearing fault detection.

In summary, the early bearing fault diagnosis method proposed in this paper—based on HO-VMD and weighted evidence fusion of current and vibration signals—achieves substantial improvements in the accuracy, precision, and recall. Its effectiveness and superiority have been further validated under small-sample conditions. The results of comparative experiments underscore that, in scenarios with limited data, the multi-source information fusion approach attains higher diagnostic accuracy than both single-signal methods and conventional AI algorithms, thereby providing a reliable technical solution for early bearing fault diagnosis in small-sample environments.

## 5. Discussion

The early fault diagnosis method for PMSM bearings proposed in this paper significantly enhances the SNR of fault signals and effectively suppresses environmental noise as well as interference from inherent motor frequencies during feature extraction. It addresses the key limitations of traditional methods, including poor reliability in early fault diagnosis and sensitivity to highly conflicting information. Consequently, the accuracy and reliability of early minor fault diagnosis are greatly improved. Nevertheless, there remains potential for further optimization and refinement of this method, which can be explored from the following perspectives:

1. The WMDST fusion method proposed in this paper combines the kurtosis of the current and vibration signals with the FNR value derived from the TKEO energy spectrum, enabling accurate identification of early faults in the bearing outer ring. Research indicates that both the current and vibration signals are highly sensitive to bearing faults and can effectively capture subtle early fault characteristics. In addition to these signals, other types of signal—such as acoustic emissions and motor temperature increase signals—also contain information relevant to bearing faults and have been utilized in related diagnostic studies. Additionally, various characteristic indicators of fault signals—including time-domain features such as the root mean square and margin factor, frequency-domain features such as the spectral energy and spectral kurtosis, and time-frequency domain features such as the wavelet packet energy and the Hilbert–Huang transform—can reflect the bearing’s health state from different perspectives. Therefore, future work may focus on integrating multiple types of motor signals and diverse characteristic indicators to develop a bearing fault diagnosis model based on multi-source information fusion, potentially further improving the comprehensiveness and accuracy of diagnostics.
2. This paper systematically verified the effectiveness and accuracy of the proposed early bearing fault diagnosis method using a PMSM experimental platform. The results demonstrate promising applicability of the method to this type of motor. Given the structural and operational differences among various motor types, future work may incorporate an asynchronous motor experimental platform for further research and analysis, aiming to broaden the method’s applicability and improve its practicality and generalizability.
3. The analysis in this paper shows that faults in the bearing inner ring and rolling elements exhibit characteristic patterns similar to those of outer race faults in both motor vibration and current signals, differing only in their characteristic frequencies and amplitudes. This suggests that the diagnostic procedure proposed herein is also applicable to faults in the inner race and rolling elements. Future research may include experimental validation under diverse bearing fault conditions to further enhance the method’s applicability and theoretical foundation.
4. Whether the weighted fused BPA function meets the preset decision rules is used in the early fault diagnosis method proposed in this paper as a criterion, achieving effective identification of early bearing faults under limited sample conditions. Compared to AI-based diagnostic methods, this approach does not rely on a large number of fault samples for model training, making it more suitable for practical scenarios with scarce data. However, the current decision-related parameters mainly depend on data from prior experiments and manual setting, lacking an adaptive adjustment capability. When the motor operating conditions or environment change, these parameters need to be readjusted, which introduces some subjectivity and limitations. Future research could explore the combination of this method with deep learning, transfer learning, or other AI technologies to optimize decision parameter selection in a data-driven manner under conditions with sufficient samples, thereby improving the adaptability and diagnostic accuracy. Additionally, AI-based online diagnosis of bearing faults is also worthy of attention. Through real-time acquisition of motor vibration signals and stator current signals, extracting fault features and feeding them into pre-trained diagnostic models, intelligent identification of the motor bearing operating status can be achieved, providing more efficient and intelligent fault monitoring solutions for use in engineering practice.

## 6. Conclusions

This paper proposes an early bearing fault diagnosis method based on HO-VMD and weighted evidence fusion of current–vibration signals to address three major challenges. First, the integration of HO-VMD decomposition with TKEO demodulation effectively enhances the early fault features while suppressing environmental noise, thereby significantly improving the SNR of fault signals. Second, weighting coefficients are assigned based on the prior reliability of each fault feature source, reducing the influence of unreliable information and thus enhancing the diagnostic reliability. Third, a weighted modification of the TDST strengthens the capability to handle conflicting information during multi-source information fusion, thus avoiding unreasonable fusion results. The experimental results demonstrate that the proposed method significantly enhances the SNR of fault signals and effectively suppresses interference from environmental noise and inherent motor frequencies during feature extraction. Moreover, it overcomes shortcomings of traditional approaches, such as poor reliability and vulnerability to highly conflicting information in early fault diagnosis. Under limited sample conditions, compared to conventional single-source methods and standard AI algorithms, the proposed approach markedly improves the accuracy and reliability of early incipient and minor bearing fault detection. Therefore, it provides a practical and robust technical solution for intelligent early fault diagnosis of bearings in industrial applications.

## Figures and Tables

**Table 1:** Bearing parameters.

Bearing Parameters	Values
Bearing model	6205
Outer raceway diameter	52 mm
Inner raceway diameter	25 mm
Ball center trajectory diameter	38.5 mm
Ball diameter	7.938 mm
Number of balls	9

**Table 2:** Effect of frequency band selection on FNR for ORFs.

Signal Type	Frequency Band	Average Noise Amplitude	Feature Noise Ratio
Vibration	0–300 Hz	−22.48 dB	2.814
	0–500 Hz	−21.77 dB	2.588
	0–1000 Hz	−19.64 dB	2.031
	0–2000 Hz	−16.25 dB	1.370
Current	0–300 Hz	−27.20 dB	4.485
	0–500 Hz	−26.90 dB	4.327
	0–1000 Hz	−23.75 dB	3.018
	0–2000 Hz	−21.53 dB	2.334

**Table 3:** Standard values of fault signals.

Fault Feature Information	Values		
	Fault	Uncertain	Healthy
Vibration—FNR	2.8	2.4	2.0
Vibration—Kurtosis	4.2	3.8	3.4
Current—FNR	4.5	3.25	2.0
Current—Kurtosis	2.8	2.7	2.6

**Table 4:** Effect of *η* threshold selection on the accuracy of ORF diagnosis.

Threshold	Correct Fault Diagnoses	Correct Healthy Diagnoses	Accuracy
0.25	50	43	93%
0.3	50	46	96%
0.35	50	49	99%
0.4	48	49	97%
0.45	45	50	95%
0.5	41	50	91%
0.6	37	50	87%

**Table 5:** Kurtosis of each IMF for fault current and vibration signals.

IMFs	Kurtosis of Current	Kurtosis of Vibration
IMF1	2.740	5.117
IMF2	2.660	7.915
IMF3	2.919	3.881
IMF4	3.786	2.534
IMF5	2.316	2.302
IMF6	1.599	2.115
IMF7		3.212

**Table 6:** Comparison of FNR for ORF.

Signal Type	Square Root of Sum of Squared Features	Average Noise Amplitude	Feature Noise Ratio
Current—Hilbert	−27.24 dB	−36.71 dB	2.968
Current—TKEO	−16.53 dB	−28.38 dB	3.911
Vibration—Hilbert	−24.25 dB	−33.28 dB	2.835
Vibration—TKEO	−11.37 dB	−22.08 dB	3.446

**Table 7:** BPA functions and fusion results for ORF.

Fault Feature Information	Evidence Source	Values		
		Fault	Uncertain	Healthy
Current—FNR	*m*_CF_	0.570	0.427	0.003
Current—kurtosis	*m*_CK_	0.005	0.798	0.197
Vibration—FNR	*m*_VF_	0.765	0.141	0.094
Vibration—kurtosis	*m*_VK_	0.699	0.180	0.121
TDST fusion		0.157	0.842	0.001
WMDST fusion		0.646	0.353	0.001

**Table 8:** Weighted BPA functions and fusion results for ORF.

Fault Feature Information	Evidence Source	Values		
		Fault	Uncertain	Healthy
Current—FNR	$m_{CF}^{’}$	0.611	0.321	0.068
Current—kurtosis	$m_{CK}^{’}$	0.558	0.341	0.101
Vibration—FNR	$m_{VF}^{’}$	0.687	0.211	0.092
Vibration—kurtosis	$m_{VK}^{’}$	0.638	0.266	0.096
TDST fusion		0.157	0.933	0.038
WMDST fusion		0.646	0.957	0.043

**Table 9:** Comparative analysis of ORF diagnosis ablation experiment results.

Diagnostic Methods	*TP*	*TN*	*FP*	*FN*	Accuracy	Precision	Recall
WMDST—Current and Vibration	50	49	1	0	99.0%	98.04%	100.0%
WMDST—Vibration	48	46	4	2	94.0%	92.31%	96.0%
WMDST—Current	40	36	14	10	76.0%	74.07%	80.0%
TDST—Current and Vibration	47	47	3	3	94.0%	94.0%	94.0%
WaveCAResNet	45	43	7	5	88.0%	86.54%	90.0%
AlexNet	42	41	9	8	83.0%	82.35%	84.0%
ConvNeXt-T	44	41	9	6	85.0%	83.02%	88.0%

## Data Availability

The data are contained within the article.
